# Predicting Clinical Efficacy of Vascular Disrupting Agents in Rodent Models of Primary and Secondary Liver Cancers: An Overview with Imaging-Histopathology Correlation

**DOI:** 10.3390/diagnostics10020078

**Published:** 2020-01-31

**Authors:** Yewei Liu, Shuncong Wang, Xiaohui Zhao, Yuanbo Feng, Guy Bormans, Johan Swinnen, Raymond Oyen, Gang Huang, Yicheng Ni, Yue Li

**Affiliations:** 1 KU Leuven, Biomedical Group, Campus Gasthuisberg, 3000 Leuven, Belgium; yyewei.liu@outlook.com (Y.L.); shuncong.wang@kuleuven.be (S.W.); yuanbo.feng@kuleuven.be (Y.F.); guy.bormans@kuleuven.be (G.B.); j.swinnen@kuleuven.be (J.S.); raymond.oyen@kuleuven.be (R.O.); 2 Shanghai Key Laboratory of Molecular Imaging, Shanghai University of Medicine and Health Sciences, Shanghai 201318, China; 3 Department of Clinical Pathology, School of Medicine, University of California, Irvine, CA 92617, USA; zhaox@uci.edu; 4 Institute of Clinical Nuclear Medicine, Renji Hospital, Shanghai Jiao Tong University School of Medicine, Shanghai 200127, China; 5 Institute of Health Sciences, Shanghai Jiao Tong University School of Medicine (SJTUSM) & Shanghai Institutes for Biological Sciences (SIBS), Chinese Academy of Sciences (CAS), Shanghai 200025, China

**Keywords:** vascular disrupting agents (VDAs), combretastatin A4 phosphate (CA4P), liver cancer, hepatocellular carcinoma (HCC), magnetic resonance imaging (MRI), contrast agents (CAs), imaging biomarker, therapeutic response

## Abstract

Vascular disrupting agents (VDAs) have entered clinical trials for over 15 years. As the leading VDA, combretastatin A4 phosphate (CA4P) has been evaluated in combination with chemotherapy and molecular targeting agents among patients with ovarian cancer, lung cancer and thyroid cancer, but still remains rarely explored in human liver cancers. To overcome tumor residues and regrowth after CA4P monotherapy, a novel dual targeting pan-anticancer theragnostic strategy, i.e., OncoCiDia, has been developed and shown promise previously in secondary liver tumor models. Animal model of primary liver cancer is time consuming to induce, but of value for more closely mimicking human liver cancers in terms of tumor angiogenesis, histopathological heterogeneity, cellular differentiation, tumor components, cancer progression and therapeutic response. Being increasingly adopted in VDA researches, multiparametric magnetic resonance imaging (MRI) provides imaging biomarkers to reflect in vivo tumor responses to drugs. In this article as a chapter of a doctoral thesis, we overview the construction and clinical relevance of primary and secondary liver cancer models in rodents. Target selection for CA4P therapy assisted by enhanced MRI using hepatobiliary contrast agents (CAs), and therapeutic efficacy evaluated by using MRI with a non-specific contrast agent, dynamic contrast enhanced (DCE) imaging, diffusion weighted imaging (DWI) are also described. We then summarize diverse responses among primary hepatocellular carcinomas (HCCs), secondary liver and pancreatic tumors to CA4P, which appeared to be related to tumor size, vascularity, and cellular differentiation. In general, imaging-histopathology correlation studies allow to conclude that CA4P tends to be more effective in secondary liver tumors and in more differentiated HCCs, but less effective in less differentiated HCCs and implanted pancreatic tumor. Notably, cirrhotic liver may be responsive to CA4P as well. All these could be instructive for future clinical trials of VDAs.

## 1. Introduction

As a potential class of anticancer therapy, vascular disrupting agents (VDAs) have been vigorously explored over the past 20 years [1,2,3]. Depriving malignant cells from blood supply, VDAs primarily attack the existing tumor vasculature to induce rapid vascular shutdown and, consequently, to result in secondary ischemic tumoral necrosis [1,2,3]. Combretastatin A4 phosphate (CA4P) is a leading small molecular VDA, which exerts potent and reversible depolymerizing effects on the tubulin cytoskeleton of endothelial cells of immature tumoral blood vessels [2,4]. Over the last two decades, following initial Phase I safety and pharmacokinetic studies [5,6], CA4P has been evaluated in Phase II-III clinical trials in combination with chemo- and/or molecular therapies in the setting of platinum-resistant recurrent ovarian cancer [7], nonsquamous non-small cell lung cancer [8] and anaplastic thyroid cancer [9], etc.

Tumor re-growth after CA4P monotherapy was considered as a fetter that prevents VDAs from being clinically used as a sole anticancer agent. In order to overcome this drawback, CA4P-engaged combinatory therapies have be explored, for instance, joining with chemotherapy [7,8,9], radiotherapy [10] and antiangiogenic therapy [11,12]. Meanwhile, a dual targeting pan-anticancer theragnostic strategy, namely OncoCiDia [13,14,15,16] has been developed. This approach adopts a radioiodinated necrosis-avid compound ^131^I-hypericin (^131^I-Hyp) as a subsequent necrosis-oriented treatment to achieve targeted internal radiotherapy to irradiate remaining viable cells and prevent tumor regrowth [14,15,16]. To date, initial clinical trials of OncoCiDia are undergoing among both veterinary [17] and human [18] patients and have shown promise [19,20,21,22].

Regarding CA4P applications in primary liver cancers, it remains unexplored in human studies likely due to a lack of relevant preclinical data and/or non-prioritized patient recruitment. To closely mimic the diverse histopathological features of hepatocellular carcinomas (HCCs) in human patients [23] who may subject to VDAs treatment, serial studies on CA4P have been conducted on the basis of chemically induced primary HCCs in rodents [24,25,26,27]. Notably, heterogeneous responses to CA4P were discovered in relation to tumor size [24], vascular geography [25,26], and cellular differentiation [24,25,26,27], which distinctly differed from the uniform pronounced tumoricidal effect in transplanted tumor models with massive central tumor necrosis embraced by a peripheral viable rim [4,15,28,29,30,31]. Therefore, this calls for noninvasive and effective evaluation methods both for proper HCC patient selection before CA4P administration, and for real-time monitoring of the drug efficacy featuring vascular shutdown in minutes to hours and tumor necrosis in hours to overnight after VDA treatment.

Magnetic resonance imaging (MRI) has been increasingly applied in researches on VDAs to morphologically and functionally investigate in vivo tumoral responses [29,30,31,32]. Superior to other clinical imaging techniques like computed tomography (CT) and ultrasonography (US), MRI enables sensitive diagnosis of early multifocal primary liver tumors in the minimum diameters about 2 mm and with nodule numbers per liver ranging from 1 to ≥ 10 in rodents [33,34,35], and allows noninvasive serial imaging follow-up at frequent time points. In addition, liver specific MRI contrast agents (CAs) may play an important role on imaging diagnosis of hepatic nodules, especially to differentiate HCCs of varying histological grades [34,35,36]. Indeed, besides imaging diagnosis and monitoring tumor growth before treatment, MRI is also capable of providing multiparametric imaging biomarkers to visualize and quantify in vivo vascular disrupting effect on hepatic malignancies [29,30,31,32]. For instance, dynamic contrast enhanced (DCE)-MRI contributes to evaluation of tumor vascular properties such as blood flow, blood volume, vascular permeability and extravascular extracellular space [37,38,39], whereas apparent diffusion coefficient (ADC) derived from diffusion-weighted imaging (DWI) helps to distinguish the cytolytic necrosis of less restricted diffusion caused by therapy from the non-responded viable tumoral cells [29,31,40,41,42]. In this context, methodologically ensured imaging-histopathology co-localization has been proven crucial to the longstanding reliability of experimental interpretations and final research conclusions [34,35].

## 2. Animal Models of Primary and Secondary Liver Cancers Usable for VDAs Studies

The liver is not only the primary site where hepatic cancer initiates, but also the most common host of metastatic disease. Thus, appropriate use of different animal models with primary and secondary hepatic malignancies is the cornerstone to perform clinically relevant evaluation of VDA-effects [26].

### 2.1. Primary Liver Cancer Model in Rats

Compared with models with intrahepatically transplanted tumor, the model of primary liver cancer generated by administration of chemical carcinogens [43] is time consuming but more fruitful. More importantly, such a hepatic carcinogenetic model is of high clinical value for mimicking human primary liver cancers that are featured by heterogeneous tumor angiogenesis, diverse histological types, various degrees of cellular differentiation and different tumor progression stages [24,25,35,36,44]. By using a complete carcinogen diethylnitrosamine (DENA) in rats, multifocal hepatocellular carcinomas (HCCs) in a full spectrum of tumor vascularity and cellular differentiation superimposed on various degrees of liver cirrhosis could be induced [35,36,43]. Etiologically, DENA induced hepatocarcinogenesis in rodents is classified as the toxics-caused chronic liver disease that evolves from liver injury, through cirrhosis and eventually into malignancy, similar to the pathologic process of chronic viral liver diseases in humans. Genetically, DENA induced mouse HCCs demonstrate high similarity to those from Myc Tgfa transgenic mice and from the poor survival group of human HCCs [45].

**Figure 1:**
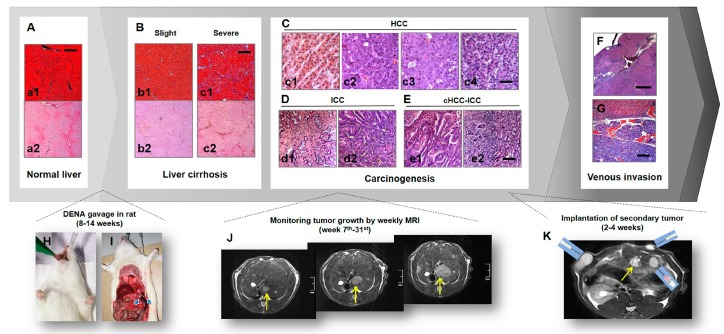
Rodent models of primary and secondary liver tumors mimicking human liver cancers. (**A**,**B**): histopathology of normal (**A**) and cirrhotic (**B**) livers (a1, b1, c1: Masson’s trichrome staining; a2, b2, c2: CD34-PAS red staining; ×100 original magnification, scale bar = 200 μm). (**C**): H&E staining of hepatocellular carcinoma (HCC) (×200 original magnification, scale bar = 25 μm). (**D**): H&E staining of intrahepatic cholangiocarcinoma (ICC) (×200 original magnification, scale bar = 50 μm). (**E**): H&E staining of cHCC-ICC (×200 original magnification, scale bar = 50 μm). (**F**): Evidence of hepatic HCC invading portal veins (H&E staining; ×50 original magnification, scale bar = 500 μm). (**G**): Tumor emboli present in hepatic vein (H&E staining; ×100 original magnification, scale bar = 100 μm). (**H**): Daily gavage of DENA in rats. (**I**): Customized plastic needle catheter precisely passing rat cardia, which effectively prevented liquid backflow and choking. (**J**): Weekly monitoring tumor growth of primary liver cancer in rats by T2-weighted MR imaging. (**K**): Implantation of secondary tumors after primary hepatoma (arrow) have developed.

In our studies, a modified DENA gavage feeding protocol was developed [25,26] (Figure 1). Briefly, DENA gavage was administrated daily in rats under instant gas anesthesia at 5–10 mg/kg/day via a prolonged flexible plastic esophagogastric tube (Fuchigami Kikai, Kyoto, Japan) [43] for eight to fourteen weeks (Figure 1H). The gavage tube was customized into this specific length of 16 cm for the purpose of precisely passing the cardia and, therefore, securely preventing liquid backflow and choking when rats were under gas anesthesia [25,26] (Figure 1I). This length fits both smaller rats like Wistar albino Glaxo/Rijswijk (WAG/Rij) strain weighting around 200 g and larger strains such as Wistar and Sprague Dawley (SD) strains weighting ≥ 400 g. Next, by monitoring tumor growth weekly with MRI, liver nodules became recognizable on T2-weighted imaging (T2WI) after seven to twenty-three weeks, and were ready for CA4P therapy after 12–31 weeks until the maximum tumor diameter in each rat reached around 1.0 cm (Figure 1J). The comparison of carcinogenesis progresses by two different doses of DENA can be found in Table 1. With a lower dose DENA administration and a prolonged exposure period, primary HCCs generally appeared higher differentiated with lower graded vascularity; meanwhile, the accompanying liver cirrhosis was also less in severity.

### 2.2. Complex Model of Primary and Secondary Liver Tumors for Comparative VDA Studies

Intraindividual comparison to secondary liver tumors in the same microenvironment of cirrhotic liver becomes achievable via further intrahepatic transplantation of other ectopic or orthotopic tumors. Take WAG/Rij rats for instance (Figure 1K), chemically induced primary HCCs and surgically implanted rhabdomyosarcoma (R1) coexisted in the same rats under the same cirrhotic background before assessment of the pharmacological behaviors of CA4P, which resulted in new insights [26].

### 2.3. Clinical Relevance of Rodent Models to Human Patients on the Use of VDAs

Chemically induced primary liver cancer model in rodents could histopathologically mimic human liver cancers at many aspects [25,26,34,35]. Increased clinical values from preclinical studies may also be realized through utilizing the similarities between animal models and cancer patients. Firstly, apart from primary HCCs (Figure 1C), intrahepatic cholangiocarcinoma (ICC) occurs as well (Figure 1D), which is the second major histological subtype of human liver cancers. As illustrated in Table 1, ICC accounts for only 2.4% of all DENA-induced primary liver tumors, while mixed hepatocellular carcinoma and intrahepatic cholangiocarcinoma (cHCC-ICC, Figure 1E) accounts for 7.7% (Table 1). Next, liver cirrhosis is generally considered as a precancerous condition on which over 80% human HCCs arise [46,47]. Under DENA exposure, hepatic cirrhosis develops in parallel with carcinogenesis (Figure 1B). In addition, HCCs tend to invade liver vasculature in patients with portal or hepatic vein tumor thrombosis (PVTT/HVTT) present in 10–40% of HCC patients at first diagnosis [48]. Similarly, HCC-thrombi were also identified in this rat model [49] (Figure 1F,G).

**Figure 2:**
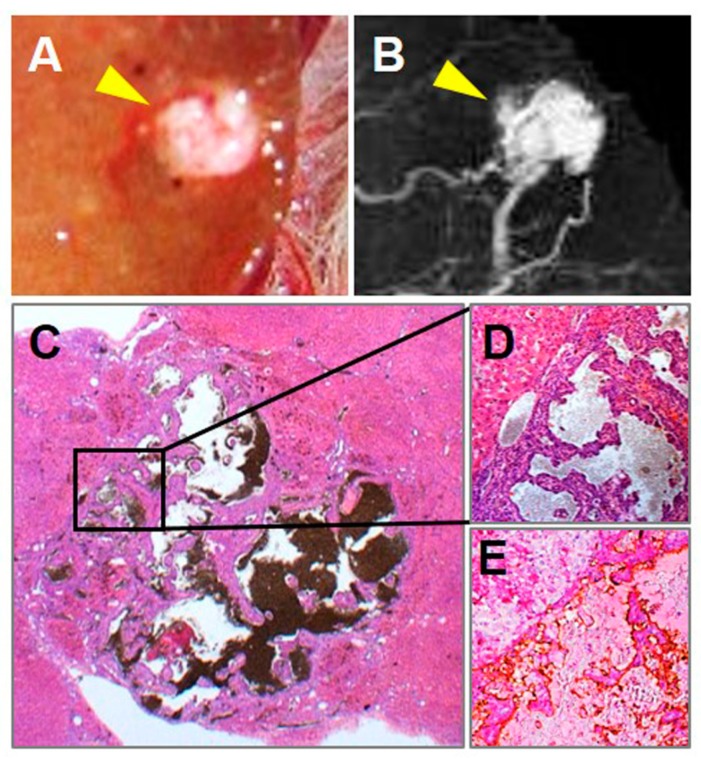
Pathological specimen, microangiographic and microscopic results of a representative angioma-like HCC. Macrographic (**A**) and (**B**) microangiographic views of a tumor-bearing liver lobe revealed a hyper-vascularized lesion (arrowhead) perfused by barium sulfate suspension. Microscopically with H&E staining, the angioma-like HCC was classified as an undifferentiated cancer type (**C**, original magnification ×12.5, scale bar = 800 µm), filled with enlarged intratumoral vascular lakes, and capsuled or circumscribed by fibrous, on liver cirrhosis background (**D**. Original magnification ×100, scale bar = 100 µm). CD34-PAS dual staining indicated that tumor blood pools were lined by positively stained endothelia (**E**. original magnification ×100, scale bar = 100 µm).

However, a type of undifferentiated liver cancer called angioma-like HCCs could be induced in this model [33,34,35,36,49], which is featured by vast vascular lakes circumscribed by fibrous capsule and sometimes with undifferentiated malignant cells floating inside vascular lakes (Figure 2). Although accounting for 7.1% of DENA-induced hepatomas in rats (Table 1), angioma-like HCC is unpopular in human HCCs.

## 3. Multiparametric MRI on Target/Receiver Selection and Efficacy Evaluation of VDA Therapy

As a powerful noninvasive imaging technique, multiparametric MRI is strong at in vivo monitoring transient tumoral responses upon VDA therapy by providing multiple functional imaging biomarkers [32]. Apart from that, MRI may be beneficial for patient screening prior to VDA therapy [25,27]. Currently, patient selection in CA4P trials is mainly based on a CA4P toxicity profile and basically focuses on exclusion standards regarding pre-existing cardiac disease [50]. In hope of achieving better responses and facilitating personalized VDA treatment, further factors such as proper tumor size, certain cancer stage, specific histological properties of vasculature and differentiation would be valuable for setting up the advanced eligibility criteria [25,27].

### 3.1. Hepatobiliary CAs

Evidences have shown that CA4P efficacy varies between primary and secondary hepatic nodules [26] and, furthermore, is reversely correlated with histological grade of primary HCCs [25]. Thus, using liver specific CAs to obtain more histological information in advance would be predictive on the outcome of VDA therapy.

**Figure 3:**
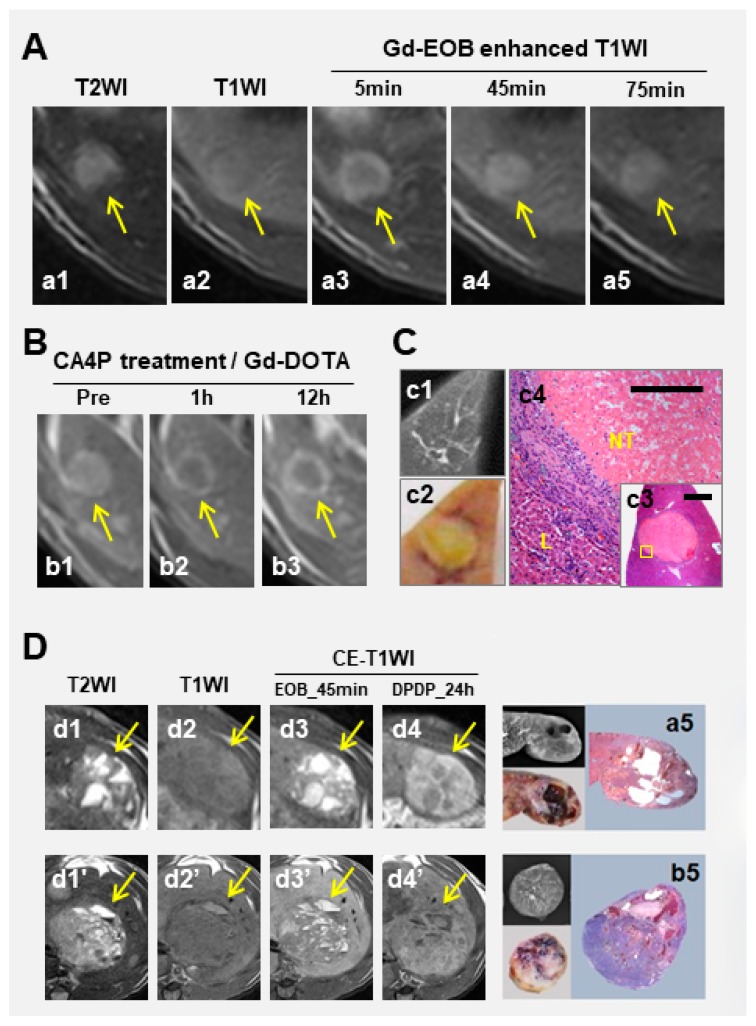
Performance of hepatobiliary contrast agents (CAs) on predicting tumoricidal effect of combretastatin A4 phosphate (CA4P) in well differentiated HCC, and on distinguish cystic foci. (**A**): A peripheral HCC lesion in the rat liver appeared hyperintense on T2WI (a1), isointense on T1WI (a2), and exhibited prolonged positive enhancement with Gd-EOB-DTPA on T1WIs throughout the hepatobiliary phase until 75 min (a3–a5), suggesting high differentiation and good response to vascular disrupting agents (VDAs). (**B**): On gadoterate dimeglumine (Gd-DOTA) enhanced T1WIs, lesion appeared hyperenhanced before CA4P treatment (b1); but central hypointense surrounded by a hyperenhanced rim at 1h (b2) and 12h (b3) post treatment, indicating vascular shutdown and consequent tumoral necrosis. (**C**): Microangiogram showed faint vascular stain (c1) in the tumor as a lesion with clear margin on macrograph (c2), which was histopathologically classified a well-differentiated HCC and proven nearly complete necrotic on both low (c3, original magnification ×12.5, scale bar = 800μm) and high (c4, original magnification ×100, scale bar = 100 μm) power microscopy (H&E staining). (**D**): Two HCCs in rats contained cysts that appeared spotted hyperintense on T2WI (d1, d1′) but hypointense on T1WI (d2, d2′), which were hyperenhanced due to Gd-EOB-DTPA accumulation in the cysts during hepatobiliary phase (d3, d3′), but without Mn-DPDP accumulation over 24 h (d4, d4′). Those cysts in HCCs (d5, d5′) showed void vasculature on microangiogram (left upper panels) and sectioned specimen (left lower panels), and H&E stained photomicrographs (right panels) revealed higher (upper panel) and lower (lower panel) cellular differentiation between two HCCs, which is in line with their degrees of Mn-DPDP enhancement (d4 vs. d4′).

Commercially available hepatic CAs such as gadoxetic acid (Gd-EOB-DTPA) [33,34,35,36] and mangafodipir trisodium (Mn-DPDP) [27,36,51] depict indiscernible hepatic nodules on T1WI in dissimilar enhancing patterns during different time windows. In hepatobiliary phase, Gd-EOB-DTPA leads to similar hypoenhancement among HCCs in all histological grades, except in a small portion of highly differentiated HCCs where prolonged positive enhancement is demonstrated [36] and favorable response to CA4P could be expected (Figure 3A–C). However, such highly differentiated HCCs only account for 2% of primary HCCs in rats [34,35,36], and the contrast between tumor and liver in most HCCs is independent of their cellular differentiation. In comparison, Mn-DPDP causes positive enhancement on T1WI, lasting for up to a few days, among primary HCCs in histological grades between I-III, and only negative enhancement in undifferentiated HCCs [27,34,35,51,52]. The tumor-to-liver contrast enhancement is reversely correlated with HCC differentiation grade [27,34,35,51,52]. Although Mn-DPDP has been withdrawn from clinics due to unsuccessful marketing, its unique value in predicting histological grades of HCCs and in differentiating primary and secondary liver tumors remains, which can now be extended to predicting responses of liver nodules to VDA treatment [27].

Notably, in terms of quantification of hepatobiliary CE-MRI, delayed enhancement of Mn-DPDP at 24 h shows better performance when the blood pool effect has vanished (Figure 3D). This is rather important in practice, because Gd-EOB-DTPA could accumulate in cysts or large vascular lakes at hepatobiliary phase around 30 min post injection (Figure 3D).

### 3.2. Nonspecific MRI CA

Gadoterate dimeglumine (Dotarem, Gd-DOTA) helps to depict viable hepatic malignancies as hyperenhanced nodules on T1WI, as liver tumors are generally hypervascularized [53]. Principally, Gd-DOTA could distinguish tumoral necrosis occurring after CA4P therapy by a nonenhanced area. However, liver tumors may remain hyperenhanced on Gd-DOTA mediated T1WI after patchy necrotic foci have already occurred. A delay in achieving peak of tumor contrast on late enhancement images after CA4P treatment could serve as a more sensitive indication of CA dumping and accumulating in necrotic tissue, similar to delayed enhancement in myocardial infarction [54].

**Figure 4:**
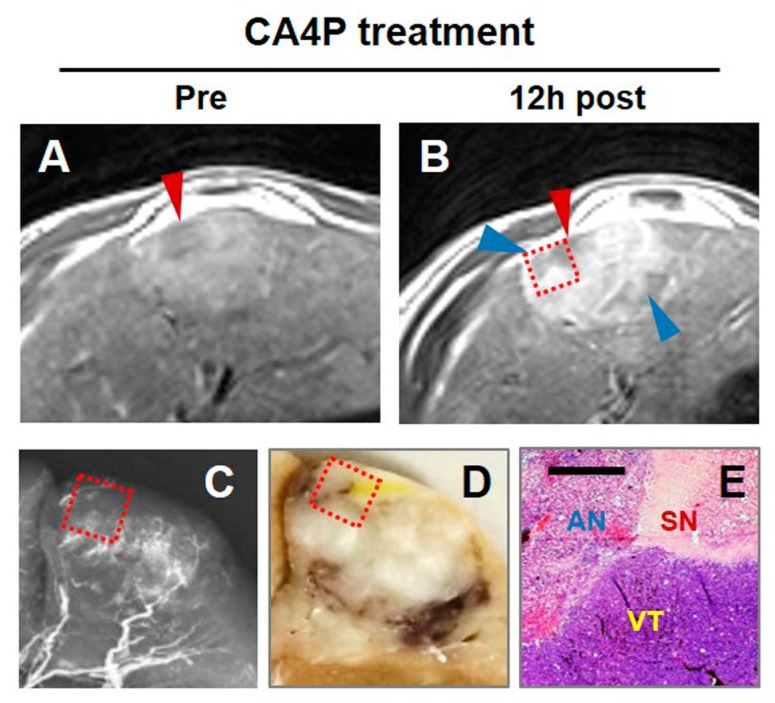
Differentiating chronic spontaneous necrosis (SN) from CA4P induced acute necrosis (AN) in an HCC. (**A**): Tumor SN (red arrowhead) existing before CA4P treatment appeared unenhanced by Gd-DOTA on T1WI. (**B**): Twelve hours after treatment, more unenhanced regions suggests CA4P-induced AN (blue arrowheads). (**C**): On microangiogram, both types of necrosis showed absent vasculature. (**D**): On photomacrograph, SN looked yellowish in contrast to the adjacent CA4P-induced hemorrhagic AN. (**E**): On photomicrograph, SN was featured by sclerotic fibrous stroma and the hyalinized degeneration, while CA4P-induced AN was identified as cell rupture, hemorrhage and inflammatory infiltration (H&E staining, ×100 original magnification, scale bar = 100 μm, VT: viable tumor).

The components of primary liver cancers are often diverse and heterogeneous including cyst, thrombus, spontaneous necrosis, etc. [25,34,35]. Identifying such nonviable areas and distinguishing them from VDA caused acute necrosis by Gd-DOTA enhanced T1WI proves crucial to precise evaluation of VDA therapeutic efficacy. In practice, spontaneous necrosis resulting from a lack of blood supply caused by rapid tumor growth [55,56] needs to be differentiated from CA4P induced acute necrosis by Gd-DOTA enhanced T1WI (Figure 4), in order not to overestimate treatment efficacy with VDAs.

**Figure 5:**
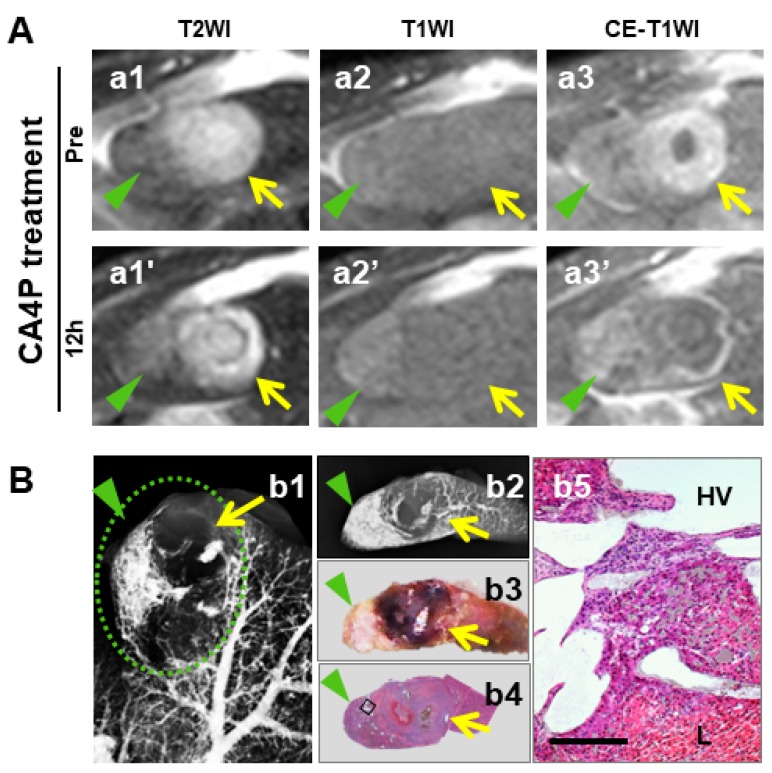
Identification of hepatic perfusion disorder (HPD) associated with focal liver tumor. (**A**): A wedge-shaped area (green arrowheads) peripheral to an intrahepatically implanted R1 tumor (yellow arrows) appeared on both T2WIs (a1, a1′) and T1WIs (a2′, a2′), and Gd-DOTA enhanced magnetic resonance imaging (MRI) (a3–a3′) independent of CA4P treatment that caused typical MRI changes of the tumor though. (**B**): Microangiographs of corresponding liver lobe (b1) and the sliced tissue block (b2) revealed a wedge-shaped area with abundant barium suspension next to the tumor, which was in line with the corresponding photomacrograph (b3) and H&E stained photomicrographs (b4, original magnification ×12.5, scale bar = 800 μm; b5, original magnification ×100, scale bar = 100 μm). The findings suggest that expanded tumor compressed local hepatic vein (HV) that was dilated and filled with barium suspension, possibly responsible for this case of HPD.

Hepatic perfusion disorders (HPD) refers to regional perfusion differences in the liver, which relates to various causes like obstructions of portal venous inflow or hepatic venous outflow, focal liver lesions and inflammatory processes [57,58]. HPD was documented in rat study with secondary liver tumor, shown as a wedge-shaped sign in peripheral hepatic segments next to the implanted tumor (Figure 5). By experiences, these areas should not be mistaken for live malignancies when VDA efficacy is analyzed.

### 3.3. Dynamic Contrast Enhancement (DCE) MRI

By bolus injection of Gd-DOTA, DCE-MRI can be further performed. Providing highly reproducible and sensitive functional information about blood volume, tissue perfusion, vascular permeability and extracellular space [29,31,59], DCE-MRI has been increasingly adopted in clinical trials of CA4P to reflect instantaneous vascular shutdown effect and thus early predict tumor responses upon VDA therapy [5,60,61,62,63].

Among the frequently used (semi-)quantitative parameters like contrast transfer coefficient (Ktrans), area under the time-signal intensity curve (IAUGC), maximal initial slope (ISpeak) and time to peak (TTP) atc. [32,59], IAUGC30 appeared the most robust against movement, regardless of whether respiratory gating is used [24,25]. IAUGC30 of HCCs at baseline was positively correlated with tumor intrinsic vascularity [25]. After CA4P treatment, IAUGC30 dropped at 1h and bounced at 12h, sensitively reflecting tumor vascular shutdown within 1h followed by reopening of tumor vasculature to varying extents [25], especially in pancreatic tumor implants [64].

### 3.4. Diffusion-Weighted Imaging (DWI)

In addition to DCE, DWI has been applied in CA4P clinical trials as well among patients with non-HCC liver metastases [12]. However, ADC calculations may be strikingly influenced by the heterogeneity of vasculature existing in primary HCCs, ranging from hypo-perfused regions (cysts, thrombus) through well perfused areas to even vascular lakes. Consequently, the transient reduction of tumor blood flow upon CA4P treatment was not always reflected by ADC_perf_ [25], while tumoral necrosis did not always correspond with changes of ADC_diff_ [25]. For similar sake, the predictive effect of ADC_diff_ on HCC differentiation reported in some literature [65] was, however, insignificant in our rat model of HCCs. ADC_perf_ decreased significantly after injection of VDAs (*p* < 0.01), indicating a decreased perfusion in tumors, which was further confirmed by post-mortem pathological analyses [24]. Consistently, K_trans_ (*p* < 0.05) and AUC_30_ (*p* < 0.01) also showed a reduction of blood flow in necrotic cancer after VDA injection [24].

## 4. Update of Antitumor Effects of CA4P in Primary and Secondary Liver Cancers

Initially, favorable responses had been expected in liver cancers upon vascular disrupting therapy, based on its known property that HCCs are among the most hypervascularized solid tumors [53]. Nevertheless, contrary to the tumoricidal effect of VDAs in secondary liver tumors, characterized by uniformly extensive central tumor necrosis rounded by a thin viable rim [15,29,30,31,32], the responsive patterns among primary liver cancers appeared much more heterogeneous. In general, CA4P could induce vascular shutdown in nearly all primary HCCs within 1h, but resulting in various degrees of tumor necrosis at 12h due to partial or complete reperfusion to primary liver cancers [25]. This phenomenon appeared even more striking between hepatic and pancreatic secondary cancers [64].

### 4.1. CA4P Dose-Related Efficacy

CA4P has a wide therapeutic window below maximum tolerated dose (MTD) in animals. At a clinically relevant dose of 10 mg/kg, CA4P functions effectively in transplanted liver tumors in rats [41]. Since a high dose of CA4P is often used in animal studies in order to achieve significant effects, we compared CA4P efficacy among primary liver cancers at two different doses (10 mg/kg and 20 mg/kg). Necrosis in primary HCCs was increased by 20% in the high dose group (unpublished data).

### 4.2. Vascularity and Differentiation of HCCs in Relation to CA4P Efficacies

In general, shortly after CA4P injection in primary HCCs, rapid vascular shutdown broadly occurred within 1h, but ended up with various degrees of tumoral necrosis, which negatively correlated with the grades of tumoral vascularity and cellular differentiation [25], which, though counterintuitive, could be translational for planning clinical trials of VDAs among HCC patients.

Several intrinsic characteristics of HCC vasculature may contribute to such tremendous variation. The first factor is whether tumor blood vessels/vascular lakes are lined up by endothelial cells, which serve as the potential target of CA4P. According to the results from immunohistochemical dual staining of CD34-PAS, all sizes of HCC’s vasculature including the vascular lakes in angioma-like HCCs are positively stained, suggesting the existing endothelia of tumoral vasculature [25]. This also supportively explains the general vascular shutdown at 1h in our studies [25,64].

Next consideration is whether all the vasculature inside HCCs responses to CA4P in the same pattern. Histopathological evidence has shown a “large-vessel-protection” sign inside CA4P induced necrotic area where HCC cells surrounding large vessels were often able to survive at the end [25]. This phenomenon might be explained by two possibilities: 1) these large blood vessels could originate from existing hepatic vessels where the normal tubulin cytoskeleton is not affected by CA4P; and 2) such wide tumor vasculature was targeted by CA4P, but only partially vascular shutdown occurred because of the enlarged lumen, while remaining blood flow could still feed the associated tumor cells.

Meanwhile, HCC differentiation also statistically correlated with CA4P efficacy. This is more likely to be an indirect correlation, since higher vascular grade corresponds simultaneously to poor HCC differentiation [25].

### 4.3. Distinct Volume-Efficacy Relation between Micro-HCCs and Macro-HCCs

Previously, a positive correlation between increasing lesion volume and better VDA therapeutic efficacy has been reported both in human studies [66,67] and in multiple murine allograft and xenograft models [68,69,70]. Consistently, this trend is implied in the primary liver cancer models when HCCs exceeded 5 mm in diameter [24]. A plausible explanation to the inferior effect of VDAs in smaller tumors is that their blood supply relies, to a large extent, on the normal blood vessels from the surrounding liver, while normal vasculature is not supposed to be targeted by VDAs [69,70]. However, nearly radical effect was paradoxically discovered in hepatic micro-HCCs [24], which is against earlier consensus that normally tumors smaller than 5 mm lack their own vasculature and are nourished by the blood diffused from their host organs [71]. This counterintuitive observation was reproduced in a rodent model with secondary liver malignancy [72]. Further studies comparing conventional and immunohistochemical (IHC) staining indicated that those micro-cancers looked avascular on H&E stained slide, but CD34 staining revealed an intratumoral network of endothelium, suggesting that such an endothelial network could be 1) functional and vital for supplying nutrients to cancer cells, and 2) vulnerable to VDA attack resulting in nearly complete tumor necrosis [72].

These new findings may imply a curative/preventive potential of OncoCiDia in early cancers especially in combination with the newly emerging supersensitive liquid biopsy techniques [73,74]. Micro-cancers of <5 mm in diameter are undetectable by current clinical imaging modalities, but are likely detectable by liquid biopsy. Imagine with a positive screening outcome, the patient may subject to a “blind” OncoCiDia episode, without knowing exactly what and where the original malignancy is, and the few residual cancer cells after CA4P in such micro-cancers can be eradicated under the full coverage of beta radiation emitted by ^131^I-Hyp with a 2–3 mm penetration distance [72]. The patient is then re-checked with a follow-up liquid biopsy weeks or months later, if the result turns out to be negative, the patient could become cured. This approach can also be applied as a post-safeguard measure to eliminate micro-metastases after other anticancer treatments such as surgery, chemo- and/or radiotherapies, thermos-ablations, etc.

On the other hand, the observed patchy necrotic foci scattered in cirrhotic liver parenchyma [24] would raise our awareness to protect hepatic tissue or function during future CA4P application in patients with underlying chronic liver diseases and consequent cirrhosis. Meanwhile, further studies need to unveil the mechanisms and potential similarity between tumor vasculature and reformatted or regenerated hepatic vasculature during cirrhosis development [24,25].

### 4.4. Superior Efficacy in Secondary Malignancies Shown by Intraindividual Comparison

Previously, tumoricidal effect of VDAs on intrahepatically implanted tumors was exhibited in the setting of healthy liver [15,29,30,31,32,64,75], while the inferior results on primary HCCs were derived from the cirrhotic liver background [24,25]. Doubts have been raised whether diverse susceptibility of liver tumors to VDA therapy is due to the intrinsic properties of tumor vasculature such as vessels density, diameter, vascular permeability, interstitial fluid pressure, etc. [76,77], or alternatively because of the dissimilar host-organ blood supplies in different implantation sites [64,75].

By using the complex liver tumor model combining primary HCCs and implanted rhabdomyosarcoma (R1) in the same rats with the same cirrhotic livers, tumoricidal effects in secondary R1 tumors and heterogeneous responses in primary HCCs were tested intraindividually [26]. This result strongly indicates that the intrinsic vasculature of individual hepatic tumors, rather than the cirrhotic versus normal liver background, fundamentally determine the observed various outcomes to CA4P [26].

**Figure 6:**
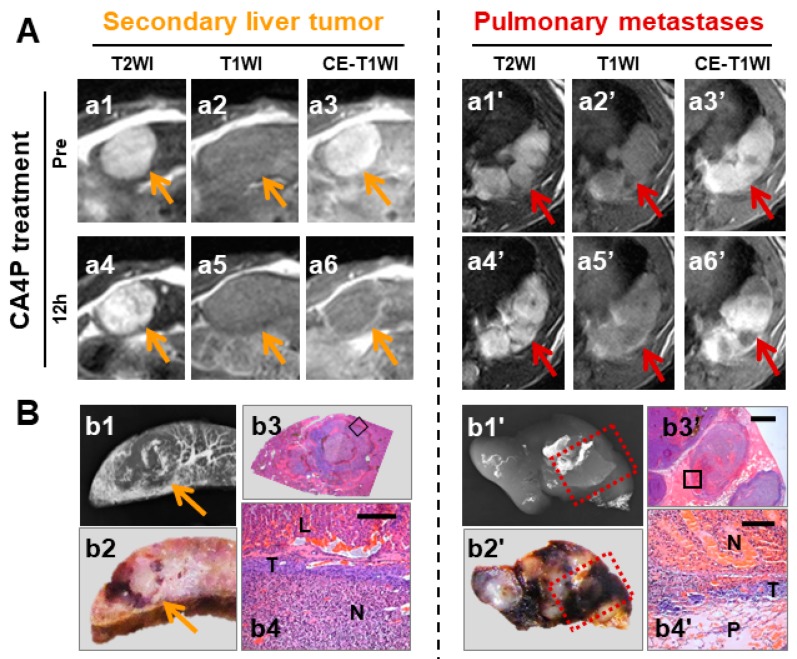
Intraindividual comparison indicating superior response in secondary liver tumor but inferior efficacy in pulmonary metastases with VDA. (**A**): In vivo MRI at three weeks after intrahepatic R1 implantation: initially, both liver R1 (orange arrows) and pulmonary metastases (red arrows) from the same tumor origin appeared hyperintense on T2WI (a1, a1′), iso- to slightly hyper-intense on T1WI (a2, a2′) compared with live parenchyma, and were enhanced on Gd-DOTA CE-T1WI (a3, a3′). Twelve hours after CA4P treatment, nearly complete necrosis occurred in hepatic R1 tumors, as reflected by extremely hyperintense on T2WIs (a4), isointense on T1WIs (a5) and an unenhanced core with a hyperenhanced rim on CE-T1WIs (a6); by contrast, regional necrosis in pulmonary metastasis was noted by hyperintensity on T2WI (a4′) and T1WI (a5′), and partial enhancement on CE-T1WI (a6′). (**B**): Corresponding microangiograms (b1, b1′), photomacrographs (b2, b2′) and photomicrographs (H&E staining; b3, b3′, original magnification ×12.5, scale bar = 800 μm; b4, b4′, original magnification ×100, scale bar = 100 μm) of liver and pulmonary R1 tumors.

Furthermore, pulmonary metastases of R1 developed simultaneously with the growth of implanted liver R1 in cirrhotic liver in the rat (Figure 6), which created the opportunity to compare VDA efficacy on secondary tumors initially from the same cluster of tumor cells but in different metastatic sites. Indeed, more extensive necrosis was identified in implanted liver R1 than in pulmonary metastases, which is likely due to the pulmonary effusion that protected metastatic R1 from being substantially deprived of nutrients during vascular shutdown by CA4P. Likewise, cancerous effusions to both abdominal and pulmonary cavities would be considered as contraindications for VDAs and OncoCiDia [15], because tumor cells may survive without blood vessels that are the target of VDAs.

## 5. Study Limitations and Practical Challenges

We recognize there were drawbacks in our experimental designs and performances. To make results more comparable to previous studies on CA4P treatment among secondary liver tumors without respiratory gating at a 1.0T or 1.5T clinical scanner [15,29,30,31,32,40,41,75], rodent models of primary liver cancers were studied with similar technical settings but at a 3.0T clinical scanner [24,25,26]. Although imaging quality of T2WI and T1WI is generally acceptable, part of the data acquired from the 3.0T scanner, which is more vulnerable to artifacts, were unanalyzable for quantifications of DCE, DWI and CE-T1WI due to the presence of motion artefacts caused by respiratory, cardiac and bowel movements. Therefore, for MRI quantitative analyses on hepatic tumors, respiratory gating is recommended [78], which is technically demanding but still possible with rodents at a clinical MRI scanner [64]. Another deficiency was a lack of quantitative DCE (Ktrans) parameter because of unavailable software package. To compensate, the semi-quantitative parameter IAUGC_30_ was alternatively applied as a sensitive detector for changes of tumor blood flow [25,26]. In the study using hepatobiliary CA [27], contrast enhancement of HCC tumors appeared fainter than that of earlier studies [33,34,35,51,52], which might result from the following reasons: 1) field-strength dependence of contrast enhancement [79] could lead to a marked decrease of R1 relaxivity of Mn-DPDP at a 3.0T magnet, compared with the previous CA studies conducted at a 1.0T or 1.5T magnet [33,34,35,51,52]; and 2) since Mn-DPDP (Teslascan^®^) was purchased and stocked in 2012 before being withdrawn from the market, oxidation of Mn2+ into Mn3+ under years of storage may weaken its relaxation property [80], hence reduced enhancement degree of HCCs on T1WI.

In terms of animal models, due to the fact that non-alcoholic steatohepatitis (NASH) has played an increasingly important role in the occurrence of HCCs due to the decreasing incidence of hepatitis and improved hepatitis management, animal models of NASH-associated HCCs may better mimic the clinical scenario nowadays [81,82]. Besides, the above-mentioned studies failed to incorporate the utilization of alpha-fetoprotein (AFP) into the multi-dimensional evaluation of tumor burden as we do in clinic for screening, diagnosis, surveillance and follow-up [83]. Demonstration of the role of AFP in patients’ selection for the OncoCiDia strategy may provide preclinical evidence for future clinical translation. At histopathological analysis, there was only circumstantial evidence indicating microvessels could be the potentially effective target of CA4P to induce necrosis in tumor parenchyma. By postmortem IHC staining, it was difficult to detect CD34 in necrotic tissue induced by CA4P because, as a transmembrane protein expressed on endothelial cells, CD34 might be destroyed along with drug-induced rupture of cell membrane. Nevertheless, the role of endothelium of tumoral vasculature in cancer ecology and VDA therapeutic response was exposed by setting and comparing treated and control groups [72].

On the other hand, due to intra- and inter-tumoral heterogeneity, experimental models of primary liver malignancies are rather therapeutically unpredictable, it was impossible to build exactly comparable primary tumors of exactly the same histopathological features, which though simulates more clinical scenarios for translational research.

## 6. Future Perspectives of VDA in Experimental Liver Cancers

Multiple radiolabeled hypericin tracers such as ^131^I-Hyp [15,16,19,20,21,22,84,85], ^123^I-Hyp [85,86,87] and 64Cu-Bis-DOTA-Hyp [88] have been investigated in different animal species of various tumors. For future clinical applications, we believe VDA-involved combinatory treatment would provide the super-effective, minimally toxic and depersonalized anticancer therapeutics. In this context, a serial preclinical and clinical trials of OncoCiDia have been ongoing [17,18], including in a particular proof-of principle experiment in animals to verify the curability of micro-cancers by OncoCiDia [72]. In addition, our imaging-based MRI-microangiography-histopathology correlation platform could be broadly applied in assessing in vivo anticancer efficacy of different types of novel agents [89].

## 7. Conclusions

Appropriate rodent models of primary and secondary liver cancers in combination with multi-parametric MRI and imaging-histopathology correlation platform are the warranties of clinically-relevant preclinical studies on tumor vascular disrupting therapy. Findings of diverse responses of CA4P in primary HCCs and the possible involvement in cirrhotic liver derived from rodent models are of translational values in guiding VDA therapy in patients with hepatic malignancies in the future. The accumulated experiences could also help candidate selection for future sequential treatment with OncoCiDia among cancer patients, in particular for possible curative or preventive management of patients with micro-cancers [72].

## Figures and Tables

**Table 1:** Induction of HCCs in rats by DENA gavage with different doses and exposure periods.

	Lower Dose [26]	Higher Dose [24,25]	*p* Value *
Animal number			
Recruited rats	16	30	
Lost rats during induction	0	5	
DENA toxicity	0	3	
Tumor hemorrhage	0	2	
Protocol of gavage			
DENA dose (mg/kg/day)	5	10	
DENA exposure period (week)	14	8	
Period of carcinogenesis (week)			
Recognizable nodule (Ø > 1mm)	16–23	7–11	
Ready for CA4P therapy (maximum Ø ≥ 8 mm)	21–31	14–22	
Tumor number	61	108	
Primary HCC lesion (89.9%)	56	96	
HCC differentiation ^§^			<0.0001
Well-differentiated (a)	15 (26.8%)	12 (12.5%)	
Moderately-differentiated (b)	26 (46.4%)	28 (29.2%)	
Poorly-differentiated (c)	13 (23.2%)	44 (45.8%)	
Undifferentiated (d)	2 (3.6%)	12 (12.5%)	
(a + b) vs (c + d)	73.2% vs 26.8%	41.7% vs 58.3%	<0.0001
Tumoral vascularity ^¶^			=0.1256
+	34 (60.7%)	41 (42.7%)	
++	13 (23.2%)	26 (27.1%)	
+++	7 (12.5%)	19 (19.8%)	
++++ (angioma-like HCCs)	2 (3.6%)	10 (10.4%)	
Primary ICC (2.4%)	0	4	
Combined HCC-ICC (7.7%)	5	8	
Liver cirrhosis	slightly	moderate - severe	

Note: * Chi-square analyses were performed to test the statistical difference of categorical variables between groups. ^§^ HCC differentiation was graded according to World Health Organization (WHO) classification of hepatic tumors. ^¶^ A grading system of tumoral vascularity for rat HCCs: vascular density similar to that in liver parenchyma (+), denser vasculature without vascular lakes (++), denser vasculature with small-sized vascular lakes (+++), and full of large vascular lakes (++++). Abbreviations: DENA: diethylnitrosamine; Ø: diameter; CA4P: combretastatin A4-phosphate; HCC: hepatocellular carcinoma; ICC: intrahepatic cholangiocarcinoma; vs: versus.
